# Nitric Oxide Affects Rice Root Growth by Regulating Auxin Transport Under Nitrate Supply

**DOI:** 10.3389/fpls.2018.00659

**Published:** 2018-05-23

**Authors:** Huwei Sun, Fan Feng, Juan Liu, Quanzhi Zhao

**Affiliations:** Laboratory of Rice Biology in Henan Province, Collaborative Innovation Center of Henan Grain Crops, College of Agronomy, Henan Agricultural University, Zhengzhou, China

**Keywords:** auxin, nitrate (NO_3_^-^), nitric oxide (NO), rice, root

## Abstract

Nitrogen (N) is a major essential nutrient for plant growth, and rice is an important food crop globally. Although ammonium (NH_4_^+^) is the main N source for rice, nitrate (NO_3_^-^) is also absorbed and utilized. Rice responds to NO_3_^-^ supply by changing root morphology. However, the mechanisms of rice root growth and formation under NO_3_^-^ supply are unclear. Nitric oxide (NO) and auxin are important regulators of root growth and development under NO_3_^-^ supply. How the interactions between NO and auxin in regulating root growth in response to NO_3_^-^ are unknown. In this study, the levels of indole-3-acetic acid (IAA) and NO in roots, and the responses of lateral roots (LRs) and seminal roots (SRs) to NH_4_^+^ and NO_3_^-^, were investigated using wild-type (WT) rice, as well as *osnia2* and *ospin1b* mutants. NO_3_^-^ supply promoted LR formation and SR elongation. The effects of NO donor and NO inhibitor/scavenger supply on NO levels and the root morphology of WT and *nia2* mutants under NH_4_^+^ or NO_3_^-^ suggest that NO_3_^-^-induced NO is generated by the nitrate reductase (NR) pathway rather than the NO synthase (NOS)-like pathway. IAA levels, [^3^H] IAA transport, and *PIN* gene expression in roots were enhanced under NO_3_^-^ relative to NH_4_^+^ supply. These results suggest that NO_3_^-^ regulates auxin transport in roots. Application of SNP under NH_4_^+^ supply, or of cPTIO under NO_3_^-^ supply, resulted in auxin levels in roots similar to those under NO_3_^-^ and NH_4_^+^ supply, respectively. Compared to WT, the roots of the *ospin1b* mutant had lower auxin levels, fewer LRs, and shorter SRs. Thus, NO affects root growth by regulating auxin transport in response to NO_3_^-^. Overall, our findings suggest that NO_3_^-^ influences LR formation and SR elongation by regulating auxin transport via a mechanism involving NO.

## Introduction

Nitrogen (N) is a major essential nutrient for plant growth (Stitt, 1999). Ammonium (NH_4_^+^) and nitrate (NO_3_^-^) are the major sources of N for plants (Kronzucker et al., 2000). Rice (*Oryza sativa* L.) is a major staple food globally. NH_4_^+^ is the main form of N in paddy soil (Wang et al., 1993). Rice roots are exposed to both NH_4_^+^ and NO_3_^-^, and are efficient at acquiring NO_3_^-^ by nitrification in the rhizosphere (Kirk and Kronzucker, 2005; Duan et al., 2007). It has been predicted that 40% of the total N acquired by rice roots grown under flooded conditions is NO_3_^-^ (Kronzucker et al., 2000; Kirk and Kronzucker, 2005). However, most previous studies on root growth and formation in response to NO_3_^-^ have focused on upland plants such as *Arabidopsis* and maize, and so further work is needed.

Plants have various mechanisms to adapt to NO_3_^-^ supply, such as plasticity of root growth (Patterson et al., 2016; Shahzad and Amtmann, 2017; Sun C. et al., 2017). Localized NO_3_^-^ supply stimulates the initiation and/or elongation of lateral roots (LRs) (Drew and Saker, 1975; Zhang and Forde, 1998; Friml et al., 2003). In *Arabidopsis*, the local stimulation of LR growth is caused by NO_3_^-^ functioning as a signal rather than as a nutrient (Zhang and Forde, 1998). Nitrate transporters, transcription factors, and micro-RNAs regulate root growth and formation in response to NO_3_^-^ (Remans et al., 2006; Vidal et al., 2010; Trevisan et al., 2011, 2012; Zhao et al., 2012, 2013; Alvarez et al., 2014; Yan et al., 2014; Huang et al., 2015). For example, *NRT1*.*1*, which encodes an NO_3_^-^ transporter, reportedly regulates the stimulatory effects of NO_3_^-^ on LR growth and development (Zhang and Forde, 1998; Zhang et al., 1999; Remans et al., 2006). *AtNRT2*.*1* is involved in the response of roots to low NO_3_^-^ supply, mainly in LR formation (Little et al., 2005; Remans et al., 2006). Knockdown of *OsNAR2*.*1*, a partner protein of the high-affinity nitrate transporter, inhibits LR formation in response to nitrate (Huang et al., 2015). NO_3_^-^ regulates root growth by posttranscriptional regulation of the NRT1.1/NPF6.3 (Bouguyon et al., 2016). NPF7.3/NRT1.5, a nitrate transporter, is involved in LR formation in *Arabidopsis* (Zheng et al., 2016). miR444a plays key roles in nitrate-dependent LR elongation and nitrate accumulation by downregulating the expression of *ANR1*-like genes in the NO_3_^-^ signalling pathway in rice (Yan et al., 2014). miR393/AFB3, an NO_3_^-^-responsive module, regulates LR density in response to external and internal N concentrations in *Arabidopsis* (Vidal et al., 2010; Vidal et al., 2013). The transcript levels of four *ANR1*-like genes, *OsMADS25*, *OsMADS27*, *OsMADS57* and *OsMADS61*, as well as *TGA1*/*TGA4* and *CPC*, are influenced by NO_3_^-^ supply and regulate root growth and formation (Yu et al., 2014; Canales et al., 2017; Sun et al., 2018). However, how plants sense external nitrate and the signal transduction system that influences root system development are remain unclear.

In addition to environmental conditions, the root growth of plant is regulated by plant hormones, such as auxin. Most auxin is synthesized in aboveground tissues by *YUCCA* family genes (Stepanova et al., 2011; Zhao, 2012) and is transported by auxin carriers, such as AUX1/LAX family (auxin-influx carriers), and ABCB/PGP and PIN family (auxin-efflux carriers) (Friml, 2003; Friml et al., 2003; Blakeslee et al., 2005; Zazimalova et al., 2010; Peret et al., 2012; Bhosale et al., 2018; Giri et al., 2018). Auxin plays a key role in root growth in response to NO_3_^-^ (Zhang et al., 1999; Zhang and Mi, 2005; Krouk et al., 2010). Localized NO_3_^-^ supply does not stimulate LR elongation in *axr4*, an auxin-insensitive mutant, which suggests that NO_3_^-^ regulates LR growth via auxin signaling pathways (Zhang et al., 1999). The NO_3_^-^ and auxin signaling pathways are linked by their effect on auxin transport through *AtNRT1*.*1* (Krouk et al., 2010). Liu et al. (2010) suggested that in LRs, NO_3_^-^-fed compartments have lower auxin levels than NO_3_^-^-free compartments, and localized NO_3_^-^ supply inhibits auxin transport from shoot to root in maize. Knockdown of *OsNAR2*.*1* decreases LR formation by inhibiting auxin transport from shoots to roots (Huang et al., 2015). However, the roles of auxin transport in regulating LR growth under NO_3_^-^ supply are more complex.

Nitric oxide (NO), as a signaling molecule, is involved in the growth and formation of the root system under NO_3_^-^ supply (Manoli et al., 2014; Trevisan et al., 2014; Sun et al., 2015; Kan et al., 2016). NO synthase-like (NOS-like) and nitrate reductase (NR) are the two key NO production pathways in plants. The NOS of plant has not been identified (Crawford, 2006; Moreau et al., 2008, 2010; Gas et al., 2009; Gupta et al., 2011), although studies that have used inhibitors of the animal NOS enzyme have demonstrated the involvement of the L-arginine pathway in the production of NO (Zhao et al., 2007). Moreau et al. (2008) suggested that *Arabidopsis AtNOS1* does not possess NOS activity, as it is a GTPase, and renamed it NO-associated enzyme (*AtNOA1*). Despite the lack of clarity on the role of *AtNOS*, the roots of *noa1* mutants (formerly *Atnos1*) have lower NO levels than WT (Guo and Crawford, 2005; Schlicht et al., 2013). In plants, the NR pathway mediates NO generation, and the nitrate concentration in roots influences the production of NO by regulating NR activity (Yamasaki et al., 1999; Meyer et al., 2005; Yamasaki, 2005). The levels of nitrate and nitrite are important determinants of NR-induced NO generation (Vanin et al., 2004). NO is a nitrate-related signal generated by the NR pathway that regulates root growth and formation (Zhao et al., 2007; Manoli et al., 2014; Trevisan et al., 2014; Sun et al., 2015). However, the mechanism by which NO regulates the root system architecture requires further investigation.

The interactions between NO and auxin in regulating root growth are closely linked (Correa-Aragunde et al., 2004; Fernández-Marcos et al., 2011; Jin et al., 2011; Chen and Kao, 2012; Sun H. et al., 2017). Application of SNP (a NO donor) and IAA/IBA (exogenous auxin) increased the lateral root (LR) formation. This effect of SNP and IBA were significantly inhibited by cPTIO (a NO scavenger)(Jin et al., 2011; Chen and Kao, 2012; Sun H. et al., 2017), suggesting that NO maybe act downstream of auxin in regulation of LR development. However, the interaction between NO and auxin in regulating root elongation is different from affecting LR formation. NO inhibited the elongation of roots by decreasing acropetal auxin transport in *Arabidopsis* and rice (Fernández-Marcos et al., 2011; Sun H. et al., 2017), suggesting that the interactions between auxin and NO in regulating root growth are complex and unclear.

Rice, an important food crop globally, is an ideal model for studying plant root growth because of its small genome size and availability of its complete genome sequence and well-characterized related mutants (Feng et al., 2002; Sasaki et al., 2002). In this study, we evaluated LR formation and the length of seminal roots (SRs) of rice and measured auxin concentrations, *DR5::GUS* activity, [^3^H] indole-3-acetic acid (IAA) transport, and NO levels under NH_4_^+^ and NO_3_^-^ supply. The results suggest that NO influences rice root growth by regulating auxin transport in response to NO_3_^-^.

## Materials and Methods

### Plant Materials

The Nipponbare and Dongjin (DJ) ecotype of rice were used in this study. *osnia2-1* and *osnia2-2* mutant lines (Sun et al., 2016) and *ospin1b-1* and *ospin1b-2* mutant lines (Sun H. et al., 2017) with the japonica cv. Dongjin ecotype were also used.

### Plant Growth

Rice seedlings were grown at day/night temperatures of 30°C/18°C under natural light in a greenhouse. Seven-days-old seedlings of uniform size and vigor were transplanted into holes in a lid placed over the top of pots (four holes per lid and three seedlings per hole). Nutrient solutions ranging from one fourth (2 days), one third (2 days), and a half (2 days) to full strength (1 day) were applied for 1 week, followed by full-strength nutrient solution for 1 week. The chemical composition of International Rice Research Institute (IRRI) nutrient solution was (mM): 2.5 (NH_4)2_SO_4_ and/or Ca(NO_3_)_2_, 0.3 KH_2_PO_4_, 0.35 K_2_SO_4_, 1.0 CaCl_2_, 1.0 MgSO_4_⋅7H_2_O, 0.5 Na_2_SiO_3_; and (μM) 9.0 MnCl_2_, 0.39 (NH_4_)_6_Mo_7_O_24_, 20.0 H_3_BO_3_, 0.77 ZnSO_4_, and 0.32 CuSO_4_ (pH 5.5).

The treatments applied were as follows: 100 nM indole-3-acetic acid (IAA), auxin transport inhibitor 300 nM N-1-naphthylphthalamic acid (NPA), 10 μM sodium nitroprusside (SNP), 25 μM Tu (tungstate), 100 μM [2-(4-carboxyphenyl)-4,4,5,5-tetramethylimidazoline-1-oxyl-3-oxide] (cPTIO), and 100 μM L-NAME (NG-nitro-L-arginine methyl ester) (Sun H. et al., 2017).

### Root System Architecture

The previous experiments (Sun et al., 2014) and the preliminary experiments suggested that the elongation of root (seminal root and adventitious root) and the lateral root (LR) number of seminal root/adventitious root were increased under NO_3_^-^ relative to NH_4_^+^. The seminal root here is the first and longest root formation from embryo and functions mainly during the early stages of rice. Therefore, SRs and the numbers of LRs on SRs were used to evaluate the effects of NH_4_^+^ and NO_3_^-^ on the root system. The length of SR was measured with a ruler. LRs were enumerated visually.

To visualize the formation of LR primordia, *pDR5::GUS*, a specific reporter that contains seven repeats of a synthetic auxin response element and reflects *in vivo* auxin levels (Ulmasov et al., 1997), were transformed into rice plants. After staining roots in β-glucuronidase (GUS) buffer for 2 h, LR primordia were enumerated using a stereomicroscope (Olympus SZX16) according to Sun H. et al. (2017). All experiments included eight replicates.

### Determination of Total N Concentration

The shoots and roots were separated from rice plants, and heated at 105°C for 30 min to kill the enzyme activities, followed by desiccation at 70°C for 48 h to a constant weight. The desiccated samples were ground into powder, and about 0.05 g of the powder was digested using 5 mL of 98% H_2_SO_4_ and about 1 mL of 30% H_2_O_2_ at 270°C for 30 min. The digested liquid was diluted to 100 mL with distilled water after cooling. The total N concentration of rice plants was analyzed using the Kjeldahl method. A 5 mL aliquot from the 100 mL digested liquid was determined by a colorimetric continuous flow analysis (Autoanalyzer 3; Bran+Luebbe, Germany) (Li et al., 2008). All experiments included eight replicates.

### Determination of IAA Levels

Indole-3-acetic acid levels of roots were determined as described previously (Lu et al., 2009). Fresh samples (0.5 g) were frozen in liquid N_2_. IAA levels were analyzed by high-performance liquid chromatography (HPLC).

To assess auxin distribution, rice plants were transformed with the *pDR5::GUS* constructs using *Agrobacterium tumefaciens* (strain EHA105). The roots were subjected to GUS staining. Stained plant tissues were photographed using a stereomicroscope (Olympus SZX16) equipped with a color CCD camera. All experiments included eight replicates.

### [^3^H] IAA-Transport

Shoot-to-root auxin transport in rice plants was assayed according to Song et al. (2013). [^3^H]IAA polar transport was assayed in root samples under NH_4_^+^ and NO_3_^-^ supply. The [^3^H]IAA solution contained 0.5 μM [^3^H]IAA (20 Ci mmol ^-1^) in 2% dimethyl sulfoxide (DMSO), 25 mM MES (pH 5.2), and 0.25% agar.

Shoot to root auxin transport in intact plants was monitored as follows. [^3^H]IAA solution (20 μL) was applied to the cut surface after rice shoots were removed at 2 cm above the junction of shoot and root. After an 18 h (overnight) incubation in darkness, two root segments, namely all the lateral root (LR) region and the root tip (RT), were weighed and incubated in 4 mL of scintillation solution. [^3^H]IAA radioactivity was detected using a multipurpose scintillation counter (LS6500; Beckman-Coulter, Fullerton, CA, United States).

The assay for acropetal (3–6 cm from the root tip) and basipetal (0–3 cm from the root tip) auxin transport was performed. [^3^H]IAA solution (3 μL) was applied to the root tip placed horizontally on a plastic film. After incubation in a humid, dark environment for 18 h (overnight), root segments were cut into two parts: (1) the distal 1 cm from the root tip and (2) the remaining 2 cm. [^3^H]IAA radioactivity was measured in the 2 cm long segments. All experiments included five replicates.

### Cortical Cell Length Analysis

Cortical cell length was analyzed as described by Jia et al. (2008). Cortical cells were visualized under a microscope (Olympus SZX16) equipped with a color CCD camera. The average cortical cell length of the maturation zone of SRs was determined using a mixture of 40–60 cortical cells at about 6 cortical cell layers (on per longitudinal section) with eight replicates in the maturation zone.

### *pCYCB1;1::GUS* Construct

The *pCYCB1;1::GUS* fusion construct was generated as described by Colón-Carmona et al. (1999), and transformed into rice plants. Plants were stained for *GUS* activity in the root tips (RTs) for 2 h at 37°C. The RTs were subjected to histochemical *GUS* staining and photographed using a microscope (Olympus SZX16) equipped with a color CCD camera. All experiments included eight replicates.

### Measurement of NO Levels in Roots

Nitric oxide was imaged by staining with 4-amino-5-methylamino-2′7′-difluorofluorescein diacetate (DAF-FM DA) under an epifluorescence microscope. The roots were soaked with 10 μM DAF-FM DA in 20 mM HEPES-NaOH buffer (pH 7.5) for 30 min in the dark. The roots were washed three times in fresh buffer and immediately visualized with a stereomicroscope (Olympus SZX16; excitation 488 nm, emission 495–575 nm) equipped with a color CCD camera. Green fluorescence intensity was quantified as described by Guo and Crawford (2005) using Photoshop software (Adobe Systems, San Jose, CA, United States). All experiments included eight replicates.

### Measurement of Nitrate Reductase (NR) Activity in Roots

Nitrate reductase activity in rice roots was analyzed by Ogawa et al. (1999). The assay mixture contained 25 mM K_3_PO_4_ buffer (pH 7.5), 10 mM KNO_3_, 0.2 mM NADH, 5 mM NaHCO_3_, and 5 μL extract in a final volume of 0.5 mL. The assays were conducted at 30°C for 15 min. The reaction was terminated by adding 50 μL of 0.5 M Zn(CH_3_COO)_2_, and excess NADH was oxidized by adding 50 μL of 0.15 mM phenazine methosulphate. The mixture was centrifuged at 10,000 × *g* for 5 min. The NO_2_^-^ level was quantified by combining 500 μL supernatant with 250 μ Lof 1% sulfanilamide prepared in 1.5 N HCl and 250 μL of 0.02% N-(1-naphthyl)ethylene-diamine dihydrochloride, and the absorbance at 540 nm was read using a spectrophotometer. All experiments included five replicates.

### Quantitative Reverse Transcription-Polymerase Chain Reaction

Total RNA was isolated from the roots of rice plants under NH_4_^+^ or NO_3_^-^ supply for 14 days. The RNA extraction, reverse transcription, and quantitative reverse transcription-polymerase chain reaction (qRT-PCR) methods were as described by Jia et al. (2011). All experiments with three replicates. The primer sets for *PINs*, *YUCCAs*, *NOA*, *NIA1*, *NIA2*, and *CYCB1;1* are listed in Supplementary Tables 1–3.

### Data Analysis

Data were pooled to calculate means and standard errors (SEs) and subjected to one-way analysis of variance (ANOVA), followed by a Ryan–Eynot–Gabriel–Welch *F*-test at *P* < 0.05 to determine the statistical significance of differences between treatments. All statistical evaluations were conducted using SPSS (version 11.0) statistical software (SPSS Inc., Chicago, IL, United States). All experiments included three independent biological replicates.

## Results

### NO_3_^-^ Regulates LR Formation and SR Elongation

**FIGURE 1:**
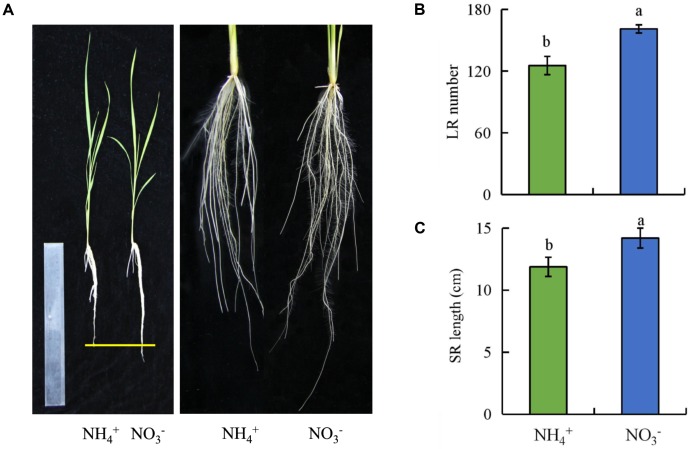
The lateral root (LR) number and seminal root (SR) length in wild-type (Nipponbare) rice seedlings. Seedlings were grown in hydroponic media containing NH_4_^+^ and NO_3_^-^ for 14 days. **(A)**, The morphology of the rice plants; **(B)**, LR number; **(C)**, SR length. Data are means ± SE and bars with different letters indicate significant difference at *P* < 0.05 tested with ANOVA.

Compared to under NH_4_^+^ supply, the number of LRs and SR length were increased by 28 and 20%, respectively, under NO_3_^-^ supply (**Figure 1**). However, the total N concentration in shoots and roots were decreased by about 20% under NO_3_^-^ relative to under NH_4_^+^ supply. These results suggest that the root growth and total N concentration of rice plants are regulated by NO_3_^-^ (Supplementary Figure 1).

### NO Is Generated by the NR Pathway and Is Involved in LR Formation and SR Elongation Under NO_3_^-^ Supply

**FIGURE 2:**
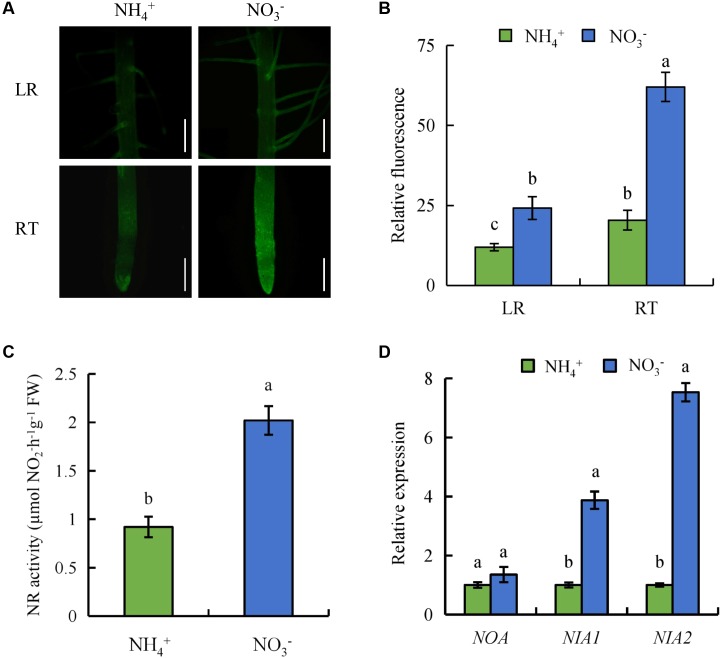
Accumulation of nitric oxide (NO), relative nitrate reductase (NR) activity, and qRT-PCR analysis of *NO-associated* (*NOA*), *NIA1*, *NIA2* genes in wild-type (Nipponbare) rice seedlings. Seedlings were grown in hydroponic medium containing NH_4_^+^ and NO_3_^-^ for 14 days. **(A,B)**, Photographs of NO production shown as green fluorescence in the lateral root (LR) and root tip (RT) **(A)**; and NO production expressed as fluorescence intensity relative to the roots **(B)**; **(C)**, Relative nitrate reductase (NR) activity in the roots; **(D)**, Relative expression of *NOA*, *NIA1* and *NIA2* in roots. Bar = 1 mm. Data are means ± SE and bars with different letters indicate significant difference at *P* < 0.05 tested with ANOVA.

To determine whether NO regulates LR formation and SR elongation under NO_3_^-^ supply, we analyzed NO-associated green fluorescence in SRs (LR region and RT) (**Figures 2A,B**). Compared to NH_4_^+^, NO-associated green fluorescence signals in RTs and the LR regions were stronger under NO_3_^-^ supply, which suggests that production of NO in roots is induced by NO_3_^-^.

**FIGURE 3:**
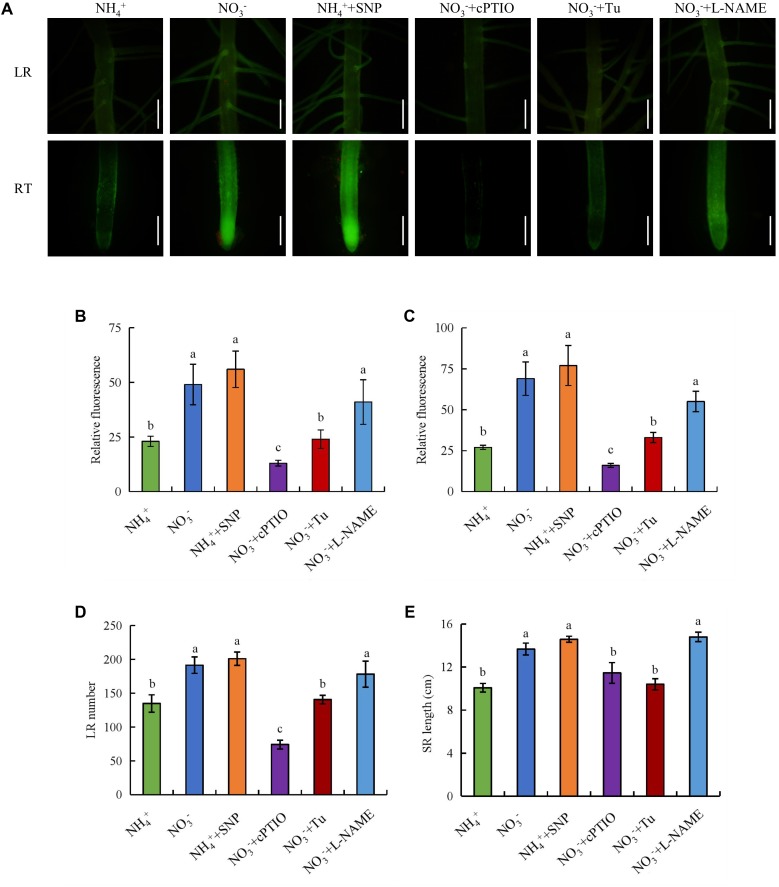
Accumulation of nitric oxide (NO) and root morphology in wild-type (Nipponbare). Seedlings were grown in hydroponic medium containing NH_4_^+^ and NO_3_^-^ in addition to SNP (10 μM), cPTIO (100 μM), Tu (25 μM) and L-NAME (100 μM) for 14 days. **(A–C)**, Photographs of NO production shown as green fluorescence in the lateral root (LR) and root tip (RT) **(A)**, and NO production expressed as fluorescence intensity relative to lateral root **(B)** and root tip **(C)**; **(D)**, Lateral root (LR) number; **(E)**, Seminal root (SR) length. Bar = 1 mm. Data are means ± SE and bars with different letters indicate significant difference at *P* < 0.05 tested with ANOVA.

We examined the functions of an NO donor (SNP) and NO scavenger (cPTIO) in root elongation and LR formation under NO_3_^-^ supply. Application of SNP under NH_4_^+^ supply significantly increased the NO-associated green fluorescence signal in SRs, the number of LRs, and the SR length to levels similar to those under NO_3_^-^ supply (**Figure 3**). However, the number of LRs, and the SR length did not respond to SNP under NO_3_^-^ supply (Supplementary Figure 2). Treatment with cPTIO under NO_3_^-^ supply markedly decreased the NO-associated green fluorescence signal, the number of LRs, and the SR length (**Figure 3**). Thus, NO production in rice roots is enhanced by NO_3_^-^ and is involved in LR formation and SR elongation.

Nitrate reductase activity in rice roots was assessed under NH_4_^+^ and NO_3_^-^ supply. NR activity increased by 119% in roots under NO_3_^-^ supply relative to NH_4_^+^ supply (**Figure 2C**). The expression of *NIA2* was significantly higher under NO_3_^-^ supply than under NH_4_^+^ supply. However, compared with *NIA2*, the expression of *NIA1* had less differences between NH_4_^+^ and NO_3_^-^. The transcript level of NO-associated (*NOA*) (a homolog of *NOA1* in *Arabidopsis*) in roots was similar under NH_4_^+^ supply and NO_3_^-^ supply (**Figure 2D**). These results suggest that NO generation is enhanced by NR rather than the NOS-like pathway under NO_3_^-^ supply.

Application of the NR inhibitor Tu (25 μM) decreased the NO-associated green fluorescence signal, the number of LRs, and the SR length under NO_3_^-^. However, treatment of rice plants with the NOS inhibitor L-NAME (100 μM) under NO_3_^-^ supply did not influence any of the parameters (**Figure 3**). These results confirm that NO is generated by NR rather than NOS-like under NO_3_^-^ supply.

**FIGURE 4:**
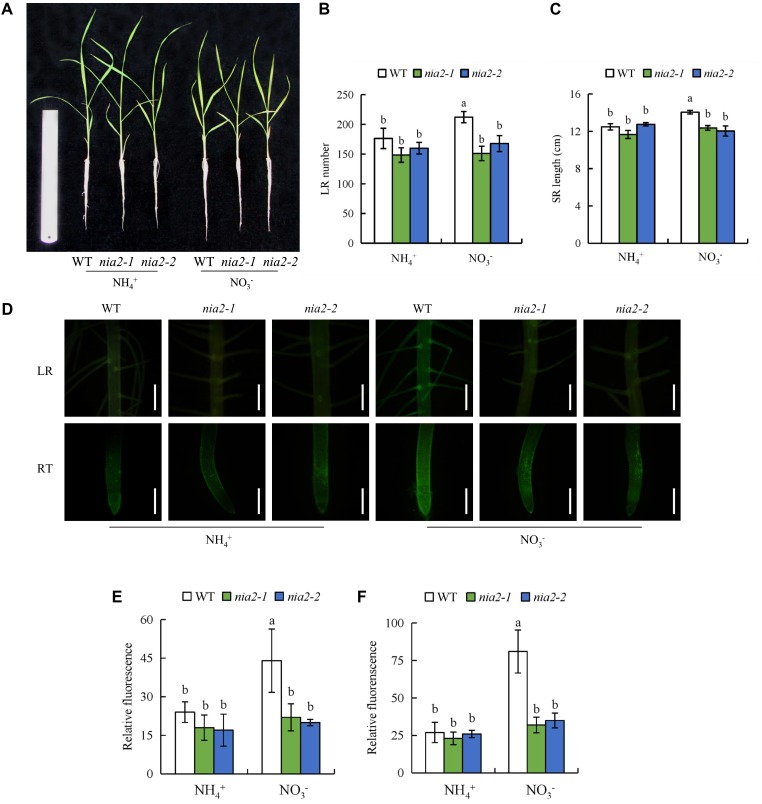
Root morphology and accumulation of nitric oxide (NO) in *nia2* mutants and wild-type (DJ) the rice seedlings. Seedlings were grown in hydroponic medium containing NH_4_^+^ and NO_3_^-^ for 14 days. **(A)**, The morphology of the rice plants; **(B)**, LR number; **(C)**, SR length; **(D)**, Photographs of NO production shown as green fluorescence in the lateral root (LR) and root tip (RT); (E-F), NO production expressed as fluorescence intensity relative to lateral root **(E)** and root tip **(F)**. Bar = 1 mm. Data are means ± SE and bars with different letters indicate significant difference at *P* < 0.05 tested with ANOVA.

The *osnia2-1* and *osnia2-2* mutant lines have reduced NR activity (Sun et al., 2016). All parameters of both *nia2* mutant lines were similar to those of WT plants under NH_4_^+^ supply, but significantly lower under NO_3_^-^ supply (**Figure 4**). Application of SNP to *nia2* mutants under NO_3_^-^ supply increased the number of LRs and the SR length to levels similar to those in the WT (Supplementary Figure 3). Moreover, treatment of WT with Tu decreased the number of LRs and SR length to levels similar to those in the *nia2* mutants (Supplementary Figure 3), confirming that NO is produced via the NIA2-dependent NR pathway under NO_3_^-^ supply.

### Auxin Levels in Roots Are Regulated by NO_3_^-^

**FIGURE 5:**
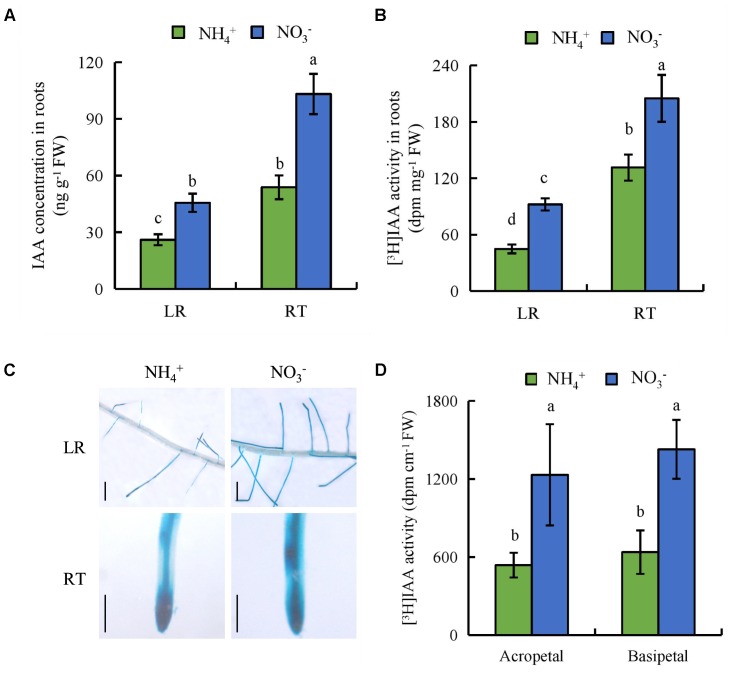
IAA concentration, [^3^H]IAA transport and histochemical localization of *DR5::GUS* activity in rice seedlings. Rice seedlings were grown in hydroponic media containing NH_4_^+^ and NO_3_^-^ for 14 days. **(A)**, IAA concentration in roots; **(B,D)** [^3^H] IAA activity in the root tip (RT) and lateral root zone (LR); **(C)**, *DR5::GUS*, a specific reporter that contains seven repeats of a highly active synthetic auxin response element and can reflect the *in vivo* auxin level. Roots were stained for GUS activity for 2 h at 37°C. Bar = 1 mm. Data are means ± SE and bars with different letters indicate significant difference at *P* < 0.05 tested with ANOVA.

We measured endogenous IAA concentrations in the LR region and RT. The endogenous IAA concentrations were 75 and 91% higher in the LR region and RT, respectively, under NO_3_^-^ relative to NH_4_^+^ (**Figure 5A**). We investigated the effects of NH_4_^+^ and NO_3_^-^ on auxin status in rice with transgenic plants transformed with the *pDR5::GUS* constructs. *DR5::GUS* activity was more widely distributed in the LR region and RT under NO_3_^-^ relative to NH_4_^+^ supply (**Figure 5C**). This was consistent with the IAA concentration results. [^3^H] IAA transport from shoots to roots was significantly higher in roots under NO_3_^-^ relative to NH_4_^+^ supply. Basipetal transport and acropetal transport of [^3^H] IAA were higher under NO_3_^-^ relative to NH_4_^+^ supply (**Figures 5B,D**). Therefore, polar auxin transport was increased under NO_3_^-^ supply.

### Auxin Is Involved in SR Elongation and LR Formation

**FIGURE 6:**
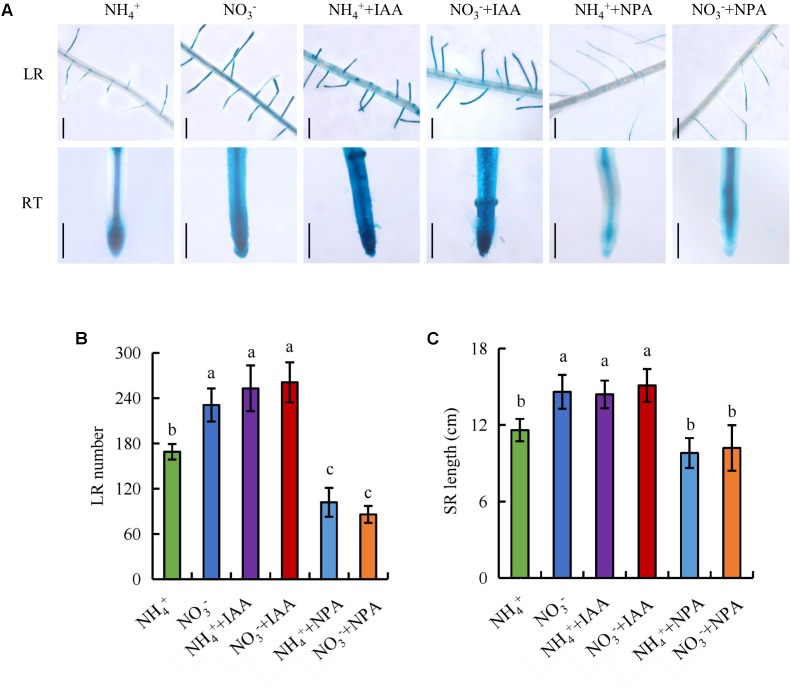
*DR5::GUS* activity and root morphology in wild-type (DJ). Seedlings were grown in hydroponic medium containing NH_4_^+^ and NO_3_^-^ in addition to IAA (100 nM) and NPA (300 nM) for 14 days. **(A)**, *DR5::GUS* activity in the lateral root zone (LR) and root tip (RT); **(B)**, Lateral root (LR) number; **(C)**, Seminal root (SR) length. Bar = 1 mm. Data are means ± SE and bars with different letters indicate significant difference at *P* < 0.05 tested with ANOVA.

We examined the number of LRs and the SR length after application of IAA and NPA (**Figure 6**). Application of IAA (100 nM) under NH_4_^+^ supply increased *DR5::GUS* expression in roots, the number of LRs, and the SR length to levels similar to those under NO_3_^-^ supply. The effects of application of IAA (100 nM) on *DR5::GUS* expression in roots and root morphology was of lesser magnitude under NO_3_^-^ supply. Treatment with NPA (300 nM) under NO_3_^-^ supply markedly decreased the *DR5::GUS* expression level in roots, the number of LRs, and SR length to levels similar to those under NH_4_^+^ supply. The effects of application of NPA (300 nM) on *DR5::GUS* expression in roots and root morphology was of lesser magnitude under NH_4_^+^ supply (**Figure 6**). These results suggest that SR elongation and LR formation are regulated by auxin transport under NO_3_^-^ supply.

### Expression of *OsPIN* Family Genes and Root Morphology of *Ospin1b* Mutants

**FIGURE 7:**
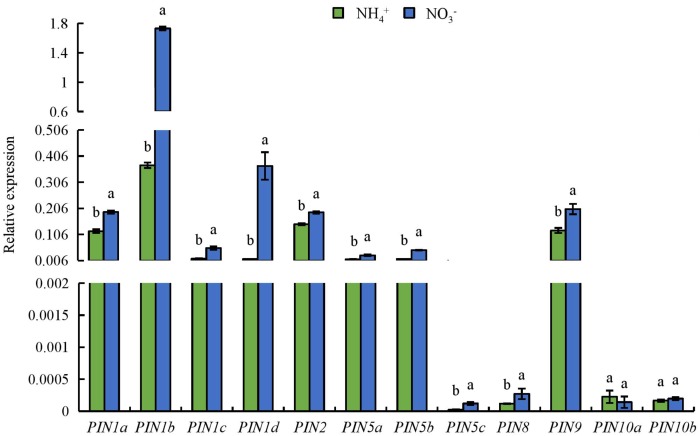
qRT-PCR analysis of *PIN* family genes in rice seedlings. Seedlings were grown in hydroponic medium containing NH_4_^+^ and NO_3_^-^ for 14 days. Relative mRNA levels were normalized for individual gene relative to Os*ACT*. Data are means ± SE and bars with different letters in the same gene indicate significant difference at *P* < 0.05 tested with ANOVA.

We analyzed the expression of the *PIN1-10* auxin transport genes in roots (**Figure 7**). Compared to under NH_4_^+^ supply, the expression levels of *PIN* genes in roots were upregulated under NO_3_^-^ supply (**Figure 7**). The expression level of *OsPIN1b* is the highest of the nine *OsPIN* genes in rice root (Wang et al., 2009; Sun H. et al., 2017). Therefore, *OsPIN1b* was used as a target gene in subsequent analyses.

**FIGURE 8:**
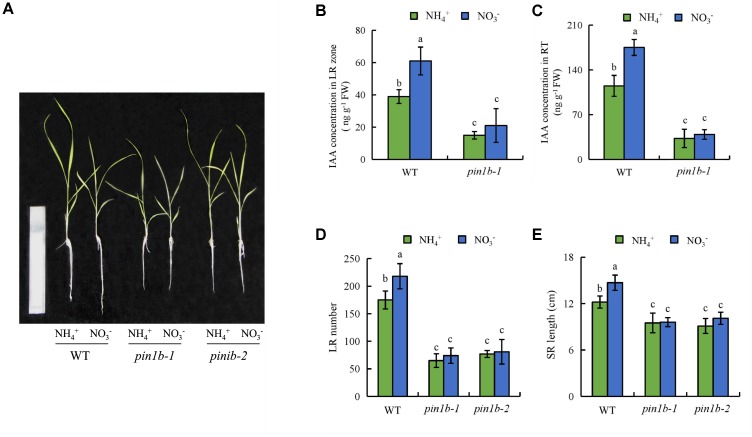
Root morphology and IAA concentration in *pin1b* mutants and wild-type (DJ) the rice seedlings. Seedlings were grown in hydroponic medium containing NH_4_^+^ and NO_3_^-^ for 14 days. **(A)**, The morphology of the rice plants; **(B,C)**, IAA concentration in the lateral root region **(B)** and root tip **(C)**; **(D)**, Lateral root (LR) number; **(E)**, Seminal root (SR) length. Bar = 1 mm. Data are means ± SE and bars with different letters indicate significant difference at *P* < 0.05 tested with ANOVA.

The *ospin1b-1* and *ospin1b-2* mutant lines have reduced auxin levels in LRs and the RT (Sun H. et al., 2017). The IAA concentration in roots of the *ospin1b-1* mutant did not differ between NH_4_^+^ and NO_3_^-^ supply (**Figures 8B,C**). The number of LRs and the SR length of the *ospin1b* mutant did not respond to NH_4_^+^ or NO_3_^-^. Compared to WT plants, the number of LRs and the SR length of the two *ospin1b* mutants were reduced under both NH_4_^+^ and NO_3_^-^ supply (**Figures 8D,E**). These findings confirm that LR formation and SR elongation are regulated by auxin polar transport under NO_3_^-^ supply.

### NO Regulates Auxin Transport Under NO_3_^-^ Supply

**FIGURE 9:**
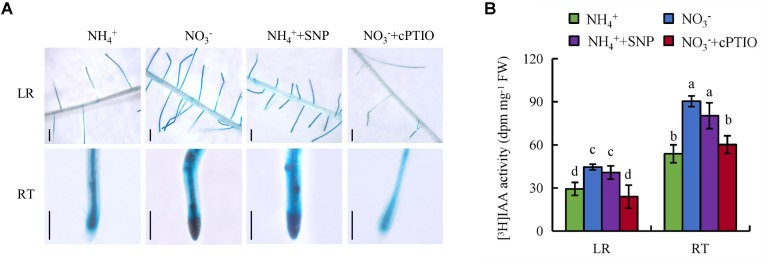
Histochemical localization of *DR5::GUS* activity and [^3^H]IAA transport in wild-type (DJ). Seedlings were grown in a hydroponic media containing NH_4_^+^ and NO_3_^-^ in addition to SNP (10 μM) and cPTIO (80 μM) for 14 days. **(A)**, *DR5::GUS*, a specific reporter that contains seven repeats of a highly active synthetic auxin response element and can reflect the *in vivo* auxin level. Roots were stained for GUS activity for 2 h at 37°C; **(B)**, [^3^H] IAA activity in root tip (RT) and lateral root zone (LR). Data are means ± SE and bars with different letters indicate significant difference at *P* < 0.05 tested with ANOVA.

Both NO and auxin are involved in regulation of root growth in response to NO_3_^-^ supply, so we investigated the effects of their interaction. Application of SNP under NH_4_^+^ supply increased *DR5::GUS* activity and [^3^H] IAA activity in roots to levels similar to those under NO_3_^-^ supply. Moreover, treatment with cPTIO under NO_3_^-^ supply decreased *DR5::GUS* expression and [^3^H] IAA activity in roots to levels similar to those under NH_4_^+^ supply (**Figures 9A,B**). However, application of IAA to roots did not affect the levels of NO in LR and RT under NH_4_^+^ condition (Supplementary Figure 6).These results suggest that NO regulates auxin transport under NO_3_^-^ supply. The expression of *YUCCA1-8* in the first leaf had no differences under NH_4_^+^ with or without SNP (Supplementary Figure 4A). However, compared with NH_4_^+^, application of SNP up-regulated the levels of *OsPIN1b* and *OsPIN1d* gene expression (Supplementary Figure 4B).

To determine the effects of duration of NO exposure for auxin buildup and root architecture change. The levels of *DR5::GUS*, LR number and SR length were examined over 16 days under NH_4_^+^ with or without SNP supply (Supplementary Figure 5). The results showed that the levels of *DR5::GUS* in LR region and RT were increased from 2 and 1 days, respectively, under SNP supply relative to application of NH_4_^+^ alone (Supplementary Figures 5A,B). Compared with sole NH_4_^+^ supply, the LR number and SR length were increased from 10 days under SNP treatment (Supplementary Figures 5C,D).

### LR Primordia Formation and Root Meristem Activity Under NO_3_^-^ Supply

**FIGURE 10:**
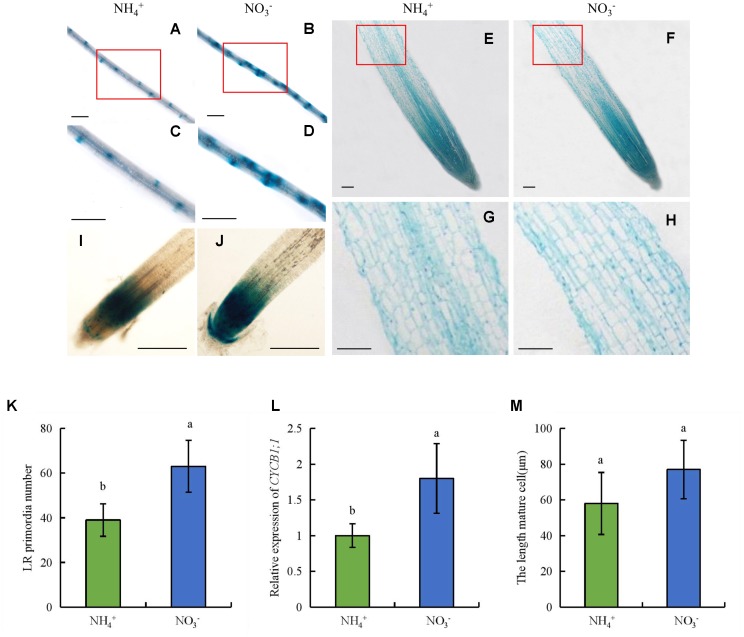
LR primordia and epidermal cell lengths in the maturity zone, expression levels of *pCYCB1;1::GUS* and *OsCYCB1;1* in the meristem zone. Seedlings were grown in hydroponic media containing NH_4_^+^ and NO_3_^-^ for 14 days. **(A–D,K)**, Lateral root primordia. Bar = 1 mm; **(E–H,M)**, Epidermal cell length of seminal root. Bar = 100 μm; **(I,J)**, Cell cycle activity of the root meristem of seminal root, as monitored by the *pCYCB1;1::GUS* reporter. Bar = 500 μm; **(L)**, The expression of *CYCB1;1* gene. Data are means ± SE and bars with different letters indicate significant difference at *P* < 0.05 tested with ANOVA.

To determine the mechanism by which NO_3_^-^ regulates LR formation and SR elongation, we enumerated LR primordia, determined the lengths of epidermal cells in the maturity zone, and assayed *CYCB1;1::GUS* activity in the RT (**Figure 10**). The number of LR primordia increased by 61% under NO_3_^-^ relative to NH_4_^+^ supply, which suggests that LR formation is dependent on LR primordia (**Figures 10A–D,K**). The lengths of epidermal cells did not differ between NH_4_^+^ and NO_3_^-^ supply (**Figures 10E–H,M**), which suggests that the promotion of root elongation by NO_3_^-^ was not due to changes in cell elongation. We used transgenic plants expressing the *pCYCB1;1::GUS* construct to assess the cyclic activity of cells in the root meristem. *CYCB1;1::GUS* activity and *CYCB1;1* expression in the root meristem were increased under NO_3_^-^ relative to NH_4_^+^ supply (**Figures 10I,J,L**). Therefore, NO_3_^-^ affected LR formation by increasing LR primordia formation and promoted root elongation mainly by increasing root meristem activity rather than the elongation of epidermal cells in the maturity zone.

## Discussion

The regulation of root elongation and formation in response to NO_3_^-^ supply is important for the growth of plants. Two examples of the plasticity of root growth and development are promotion of root elongation and LR growth under NO_3_^-^ supply. In upland species such as maize and *Arabidopsis*, the root length is increased under NO_3_^-^ supply (Liu et al., 2013; Manoli et al., 2014) and localized NO_3_^-^ supply stimulates LR elongation (Zhang and Forde, 1998; Friml et al., 2003). In rice, localized NO_3_^-^ supply may stimulate LR elongation relative to no NO_3_^-^ supply (Wang et al., 2002). In this study, compared to NH_4_^+^, NO_3_^-^ supply increased the SR length and stimulated the formation of LRs (**Figure 1**), which suggests that the functions of NO_3_^-^ in regulating rice root growth and development are similar in maize and *Arabidopsis*.

Several lines of studies suggested that NO had two strategies in plants response to NO_3_^-^ supply. Firstly, NO as a signaling molecule functions in the regulation of root growth and formation in plants under NO_3_^-^ condition (Manoli et al., 2014; Sun et al., 2015). Manoli et al. (2014) suggested that the NO_3_^-^-induced increase in root length is dependent on the NO signaling pathway. Sun et al. (2015) showed that NO is induced by partial nitrate nutrition (PNN) and is involved in LR formation in rice. Secondly, NO enhanced N uptake by increasing the expression of N transport genes under PNN (Sun et al., 2015). In this study, application of SNP increased the SR length and LR number under NH_4_^+^. Treatment with cPTIO under NO_3_^-^ supply decreased the SR length and the number of LR (**Figure 3**), These results confirm that NO is involved in LR formation and SR elongation in the presence of NO_3_^-^. The concentrations of total N in rice plants were decreased under NO_3_^-^ relative to NH_4_^+^ (Supplementary Figure 1), suggesting NH_4_^+^ is the main N source for rice. NO was induced by PNN condition and NO could enhance the N uptake in rice (Sun et al., 2015). In this study, NO production was induced by NO_3_^-^ maybe a strategy for rice plants to obtain more N.

NOS-like and NR pathways participated in NO production in plants (Wilson et al., 2008). In *Arabidopsis*, the gene of *AtNOS1* did not regulate NOS activity, therefore, it was renamed NO-associated enzyme (*NOA1*) (Moreau et al., 2008). The NO levels were significantly decrease in the root of *noa1* mutant (formerly *Atnos1*) relative to WT plants (Guo and Crawford, 2005). Besides *NOA1*-dependent pathway, *NIA1* was involved in NR-regulated NO production in plants (Bright et al., 2006; Zhao et al., 2009). *NIA2* expression is higher than that of *NIA1* (Fan et al., 2007; Sun et al., 2015). Sun et al. (2015) reported that the NO generated by *NIA2*-dependent NR increases LR formation in rice. In this study, NR activity and *NIA2* expression were significantly higher under NO_3_^-^ supply relative to NH_4_^+^ supply. Moreover, the regulation of SR elongation and LR formation by NO_3_^-^ was inhibited by Tu (NR inhibitor) but not by L-NAME (NOS inhibitor) (**Figures 2D**, **3**), which suggests that NO regulated root growth and formation under NO_3_^-^ supply main via the NR pathway. The changes in the root morphology and NO-associated green fluorescence signal of *nia2* mutants were little affected by NO_3_^-^ (**Figure 4**). This suggests that NO is produced by the NR pathway rather than the NOS-like pathway and is involved in regulation of root growth under NO_3_^-^ supply.

Auxin distribution in the LR region is regulated by auxin transport, and auxin controls LR initiation and elongation in response to NO_3_^-^ supply (Grieneisen et al., 2007; Vanneste and Friml, 2009; Krouk et al., 2010; Song et al., 2013). Application of a low concentration of NO_3_^-^ affects LR growth by regulating auxin transport (Krouk et al., 2010). Liu et al. (2010) reported that local application of NO_3_^-^ reduces acropetal and basipetal transport compared to N-free treatment, and decreases auxin distribution in the LR region to a level more suitable for LR elongation in maize. Song et al. (2011, 2013) found that auxin synthesis and auxin transport from shoot to root are higher under (PNN treatment relative to application of NH_4_^+^ alone in a high-NO_3_^-^-response rice cultivar. The polarity of auxin transport is determined by the asymmetric localisation of the *AUX1* and *PIN* auxin influx and efflux facilitators (Kramer, 2004). PIN proteins are the main auxin efflux carriers in plants (Friml et al., 2003; Wisniewska et al., 2006). Song et al. (2013) reported that *PIN5b* expression is upregulated under PNN relative to NH_4_^+^ supply. In *Arabidopsis*, *PIN2* expression is upregulated in roots under NO_3_^-^ supply compared to NH_4_^+^ (Liu et al., 2013). In this study, the auxin levels in LR and RT were higher under NO_3_^-^ relative to NH_4_^+^ supply (**Figures 5B,D**), which suggests that the auxin distribution in roots is regulated by NO_3_^-^ supply. [^3^H] IAA transport and *PIN* family gene expression were increased under NO_3_^-^ relative to NH_4_^+^ supply (**Figures 5B,D**, **7**), which suggests that *PIN* genes are involved in auxin transport under NO_3_^-^ supply.

NO and auxin help regulate root growth and formation (Jin et al., 2011; Chen and Kao, 2012). NO acts downstream of auxin in regulating lateral root formation (Chen et al., 2010; Jin et al., 2011; Cao et al., 2017) and affects root elongation by regulating polar auxin transport (Fernández-Marcos et al., 2011). In rice, NO functions downstream of auxin in regulating LR formation but inhibits elongation of root by decreasing auxin levels in root tips under Fe deficiency (Sun H. et al., 2017). However, Manoli et al. (2016) found that the NO-mediated root apex responses to NO_3_^-^ are regulated by auxin in maize. These results suggest that the interactions between auxin and NO in regulating root growth are complex. In this study, application of SNP under NH_4_^+^ supply increased the auxin levels in roots, and treatment with cPTIO under NO_3_^-^ supply decreased the auxin levels in the roots (**Figure 9**). Thus, NO is involved in NO_3_^-^-regulated auxin transport in roots. However, treatment with IAA did not affect the level of NO in roots under NH_4_^+^ supply, consistent with the previous report by Sun H. et al. (2017). These results suggested that NO maybe act upstream of auxin in regulating root growth and formation. The expression of *PIN1b* and *PIN1d* in roots were up-regulated under SNP supply relative to application of NH_4_^+^ alone. However, the expression of *YUCCAs* in the first leaf had no changes between NH_4_^+^ and NH_4_^+^ in addition to SNP (Supplementary Figure 4), suggesting that NO increased auxin levels in root mainly by regulating auxin transport but not auxin synthesis. Compared to WT, roots of the *pin1b* mutant had lower auxin levels, fewer LRs, and shorter SRs (**Figure 8**). Moreover, the root morphology of the *pin1b* mutant had less changes between NH_4_^+^ and NO_3_^-^ (**Figures 8D,E**). Therefore, NO_3_^-^ affects root growth by regulating root auxin transport via a mechanism involving NO. And these results suggest that the interactions between auxin and NO in regulating root growth in response to NO_3_^-^ supply are different from Fe deficiency.

Lateral root formation is dependent on LR primordia initiation under NO_3_^-^ supply (Song et al., 2013; Sun et al., 2015). In this study, the number of LR primordia was higher under NO_3_^-^ supply compared to NH_4_^+^. Root length depends on two basal formation processes: cell division in the RT meristem and the length of root cells in the maturity zone (Scheres et al., 2002). The activity of meristematic cells in the root meristem affects root elongation (Blilou et al., 2005). NO_3_^-^ supply increases root meristem activity by regulating the expression of *CYCB1;1* in *Arabidopsis* (Liu et al., 2013). In this study, NO_3_^-^ supply increased *pCYCB1;1::GUS* construct and *CYCB1;1* expression levels in the RT but did not affect the length of mature cells (**Figure 10**). These findings suggest that SR elongation is regulated by increasing cell division in the root meristem zone under NO_3_^-^ relative to NH_4_^+^ supply.

## Conclusion

In conclusion, NO is generated mainly by the NR pathway and induces LR formation and SR elongation by regulating auxin transport in the presence of NO_3_^-^. NO_3_^-^ influences LR formation by increasing the number of LR primordia, and root elongation by increasing root meristem activity.

## Author Contributions

HS and FF performed the experiments and wrote the paper, JL analyzed the data, QZ designed the experiment.

## Conflict of Interest Statement

The authors declare that the research was conducted in the absence of any commercial or financial relationships that could be construed as a potential conflict of interest.
