# Corrigendum: Nitric Oxide Affects Rice Root Growth by Regulating Auxin Transport Under Nitrate Supply

**DOI:** 10.3389/fpls.2019.01123

**Published:** 2019-09-17

**Authors:** Huwei Sun, Fan Feng, Juan Liu, Quanzhi Zhao

**Affiliations:** Laboratory of Rice Biology in Henan Province, Collaborative Innovation Center of Henan Grain Crops, College of Agronomy, Henan Agricultural University, Zhengzhou, China

**Keywords:** Auxin, nitrate (NO_3_^-^), nitric oxide (NO), rice, root

In the original article, there was a mistake in **Supplementary Figure 5A** as published. The image of NH_4_^+^+SNP treatment at 1d was incorrect. The corrected **Supplementary Figure 5A** appears below. The authors apologize for this error and state that this does not change the scientific conclusions of the article in any way. The original article has been updated.

