# Supplementary material for: Nitric Oxide Affects Rice Root Growth by Regulating Auxin Transport Under Nitrate Supply
Source: Front Plant Sci. 2018 May 23;9:659. doi: 10.3389/fpls.2018.00659 (PMC5974057; doi:10.3389/fpls.2018.00659)
Supplement: Supplementary file 1 [file Data_Sheet_1.pdf]

Supplementary Table 1 The primers for qRT-PCR of *PIN* family genes

Gene	Primer sequence
OsPIN1a	5'-TCATCTGGTCGCTCGTCTGC-3'
	5'-CGAACGTCGCCACCTTGTTC-3'
OsPIN1b	5'-TGCACCCTAGCATTCTCAGCA-3'
	5'-CCCTCCTCCCAAATTCTACTT-3'
OsPIN1c	5'-CCGTCAGGTTCCTCGTGGGT-3'
	5'-TCACGGCTGTGCTCAGAATG- 3'
OsPIN1d	5'-GATTCCGACGTCGTCTCGCTCG-3'
	5'-GTCGGGTTCCGCGACGACTGCA-3'
OsPIN2	5'-CAACACCTACTCCAGCCTC-3'
	5'-TGGACCAGTCAAGAACCTC-3'
OsPIN5a	5'-GGGGCTGGTGCTAAAGTTCG-3'
	5'-TGAGGTAGGGCTGCCTGTATG-3'
OsPIN5b	5'-GGGCAGCAGGAGAGGGTGATAG-3'
	5'-GAATCGGCAGAGAGATCAATGT-3'
OsPIN5c	5'-CTTCACCGCCGACCAGTGCGAC-3'
	5'-GTGATGCACCACGAGAACCCGC-3'
OsPIN8	5'-GTTCCACTATATGTAGCTATGATAC-3'
	5'-CAGTCAAACTTCTCTGCACAGC-3'
OsPIN9	5'-GATACAAGATAGCGTCGTTCTC-3'
	5'-ATGATGTCTGCGTGGACCT-3'
OsPIN10a	5'-GTTGGATTGAGATAGGCTGAGGAG-3'
	5'-ATGGCGACGAAGCGGTTGAT-3'
OsPIN10b	5'-TCCGATGCAGGGTTAGGC-3'
	5'-AGGATGGTAGCGTGGAGGTT-3'
OsACTIN	5'-CAACACCCCTGCTATGTACG-3'
	5'-CATCACCAGAGTCCAACACAA-3'

Supplementary Table 2 The primers for qRT-PCR of *YUCCA* genes

Gene	Primer sequence
OsYUCCA1	5'-AGGTGTTGGTCGTGGGATGCG-3'
	5'-GCGATGCCGAACGTGGATAGA-3'
OsYUCCA2	5'-TATGGATCGGCAACCATTTGA-3'
	5'-CGCTGGGAAGACTGTCCTTGT-3'
OsYUCCA3	5'-GGAAGCGTGTTCTCGTTGTTG-3'
	5'-ACATTGACAGCCCAAAGGTGG- 3'
OsYUCCA4	5'-CCTCGACCTCTGCAACCACAATG-3'
	5'-CGACAACAGGAGTACCAGCCAATC-3'
OsYUCCA5	5'-GTCAGCCTCGACCTCTGCAACA-3'
	5'-TGGGAAACCACTTGAGAAGGAACAC-3'
OsYUCCA6	5'-GGATACCAAAGCAACGTCCCC-3'
	5'-TGAAGCCAACAGAGTAGAGCCCTG-3'
OsYUCCA7	5'-ACCGGCTACCGCAGCAATGTG-3'
	5'-CGTACAGCCCCGACTCACCCT-3'
OsYUCCA8	5'-GAGATGTGCCTGGACCTCTGC-3'
	5'-GTGTCTCCCAGCACCATCCTT-3'

Supplementary Table 3 The primers for qRT-PCR of *NOA*, *NIA1*, *NIA2*, *CYCB1;1* genes

Gene	Primer sequence
OsNOA	5'-TGCTTCTGTGGTTGGGAC-3'
	5'-TCTAAGGGCACGGTGTTT-3'
OsNIA1	5'-CCAATTCTTTCATCGTGTTCT-3'
	5'-CATGCAGCATTTCGTTTCT-3'
OsNIA2	5'-ACTGGTGCTGGTGCTTCTGG-3'
	5'-CGGCTGGGTGTTGAGGGACT-3'
OsCYCB1;1	5'-CACGTCGACTATAGACTAAGCCATTGAGGCGTAT -3'
	5'-AAAGGTACCAGAGCTGATCTCGATGACATGCTCGG-3'

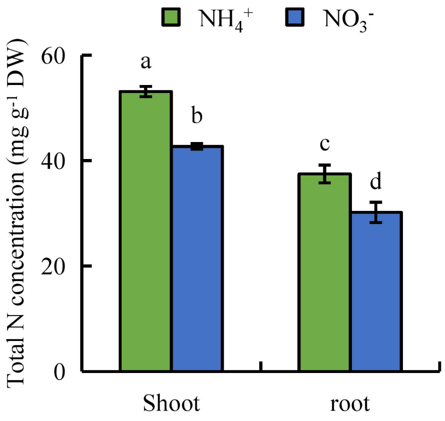

Supplementary FIGURE 1 Effect of NH_4^+ and NO_3^- availability on total nitrogen (N) concentrations in the shoot and root of wild-type (Nipponbare) rice plants. Seedlings were grown in a hydroponic media containing NH_4^+ and NO_3^-. Data are means ± SE and bars with different letters indicate significant difference at $P < 0.05$ tested with ANOVA.

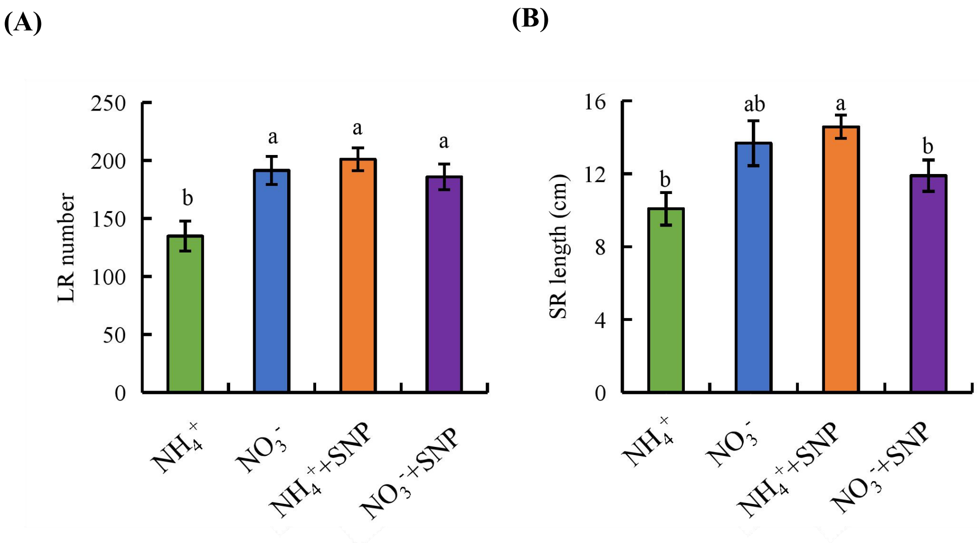

Supplementary FIGURE 2 Lateral root (LR) number and seminal root (SR) length in wild-type (Nipponbare) rice seedlings. Seedlings were grown in hydroponic medium containing NH_4^+ and NO_3^- in addition to SNP (10 μM) for 14 days. (A), Lateral root (LR) number; (B), Seminal root (SR) length. Bar=1mm. Data are means ± SE and bars with different letters indicate significant difference at $P < 0.05$ tested with ANOVA.

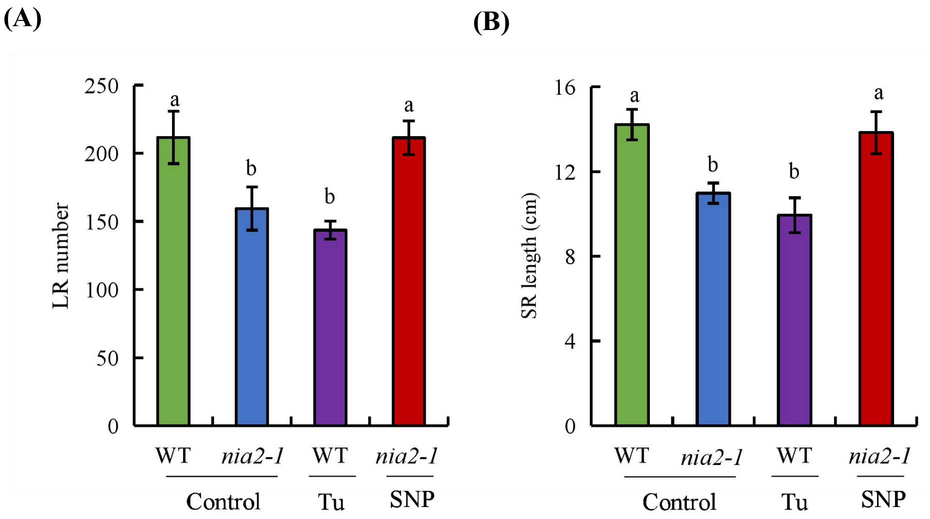

Supplementary FIGURE 3 Lateral root (LR) number and seminal root (SR) length in *nia2* mutant and wild-type (DJ) rice seedlings. Seedlings were grown in a hydroponic media containing NH_4^+ and NO_3^- in addition to SNP (10 μM) and Tu (25 μM) for 14 days. (A), Lateral root (LR) number; (B), Seminal root (SR) length. Data are means ± SE and bars with different letters indicate significant difference at $P < 0.05$ tested with ANOVA.

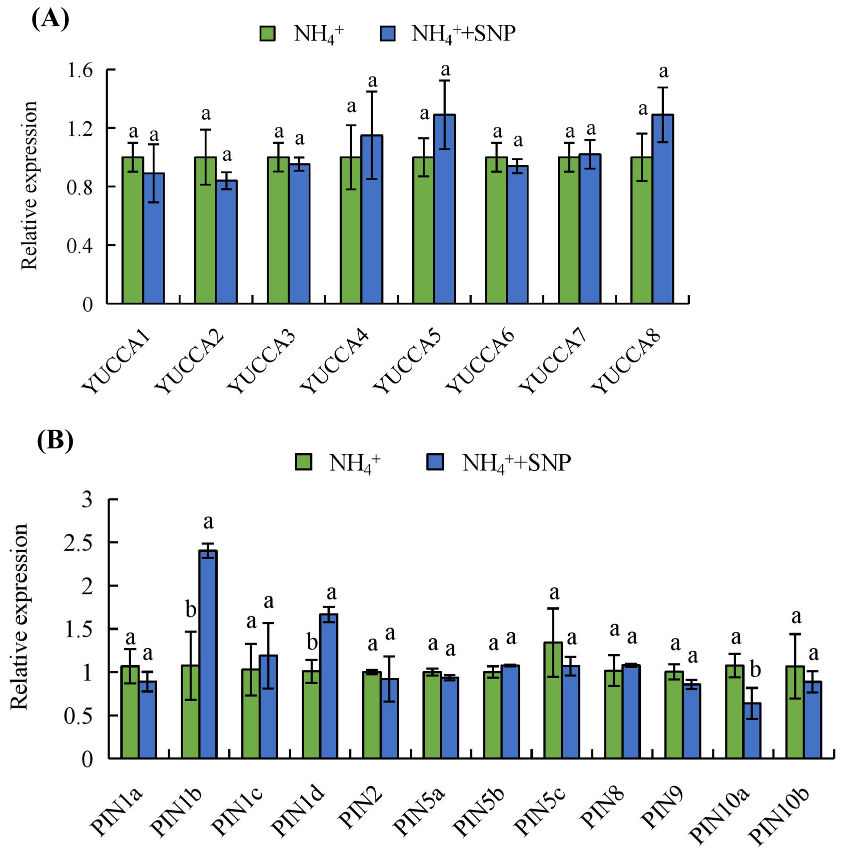

Supplementary FIGURE 4 qRT-PCR analysis of *YUCCA* and *PIN* family genes in rice seedlings. Rice seedlings were grown in hydroponic media containing NH_4^+ and NO_3^- in addition to SNP (10 μM) for 14 days. (A), The expression of *OsYUCCA* genes in the first leaf. (B), The expression of *OsPIN* genes in roots. Relative mRNA levels were normalized for individual gene relative to Os*ACT*. Data are means ± SE and bars with different letters in the same gene indicate significant difference at $P < 0.05$ tested with ANOVA.

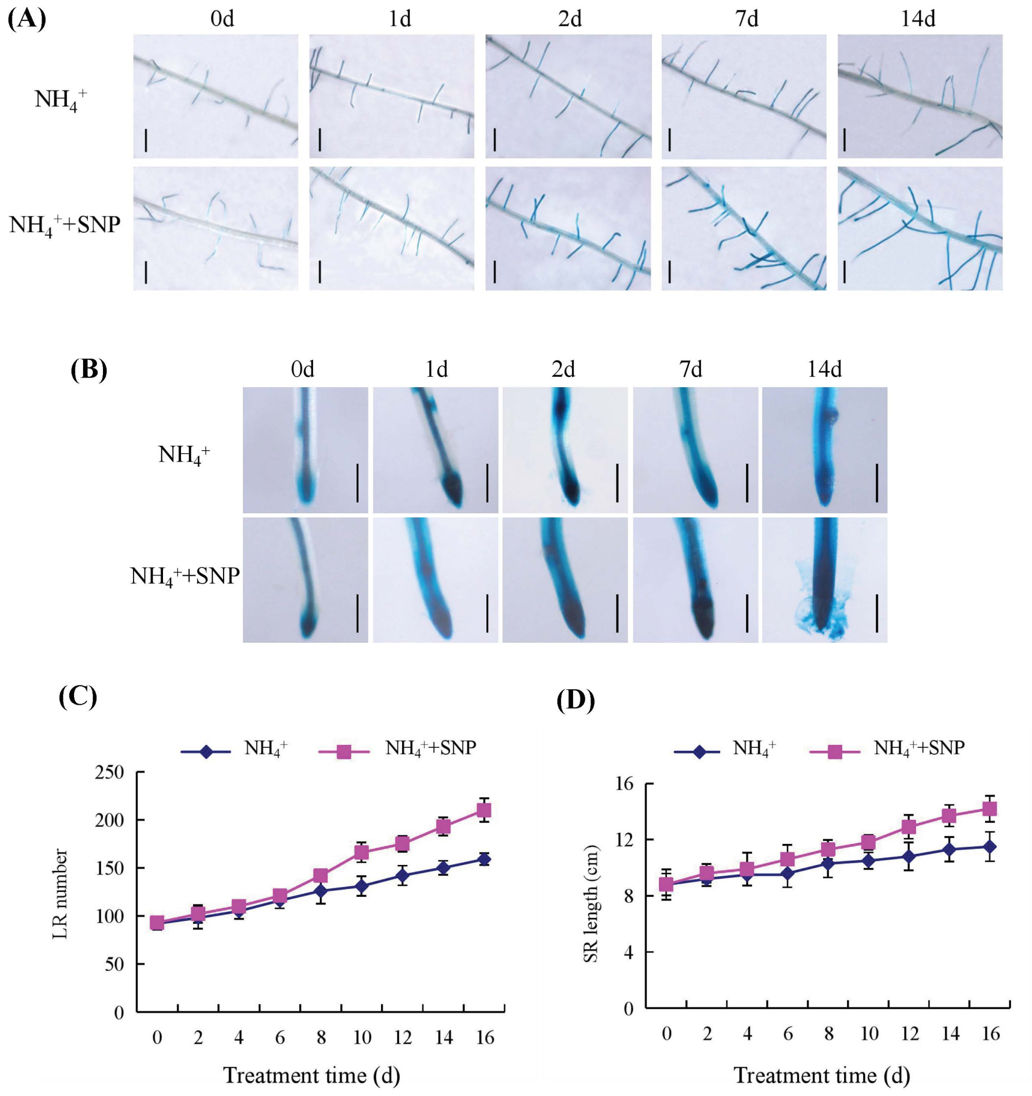

Supplementary FIGURE 5 Histochemical localization of *DR5::GUS* activity and Lateral root (LR) number and seminal root (SR) length in rice seedlings. Rice seedlings were grown in hydroponic media containing NH_4^+ in addition to SNP (10 μM) for 16 days. (A), *DR5::GUS*, a specific reporter that contains seven repeats of a highly active synthetic auxin response element and can reflect the in vivo auxin level. Roots were stained for GUS activity for 2h at 37°C. (A), Lateral root region. Bar=2mm. (B), Root tip. Bar=1mm. (C), Lateral root (LR) number. (D), Seminal root (SR) length. Data are means ± SE and bars with different letters indicate significant difference at $P < 0.05$ tested with ANOVA. d=days.

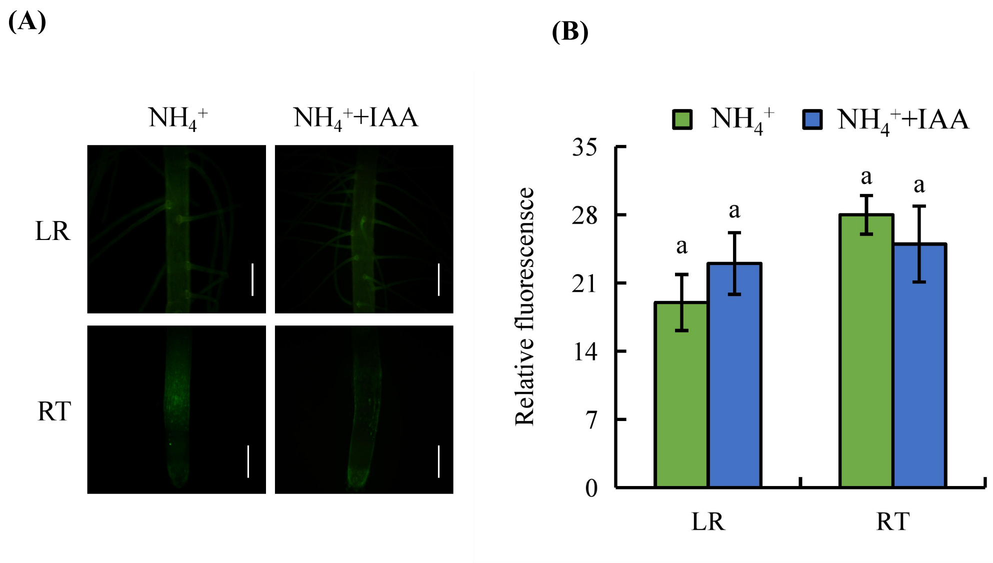

Supplementary FIGURE 6 Accumulation of nitric oxide (NO) in wild-type (Nipponbare) the rice seedlings. Seedlings were grown in hydroponic medium containing NH_4^+ with or without IAA (100 nM) for 14 days. (A), Photographs of NO production shown as green fluorescence in the lateral root (LR) and root tip (RT). (B), NO production expressed as fluorescence intensity relative to LR and RT. Bar=1mm. Data are means ± SE and bars with different letters indicate significant difference at $P < 0.05$ tested with ANOVA.
